# Why catch when you can throw? A framework for tagging animals without capture or restraint

**DOI:** 10.1098/rsos.250139

**Published:** 2025-07-16

**Authors:** Rory Wilson, James Redcliffe, Mark Holton, Phillip Hopkins, Victoria Thomas, Frank Narve Rosell, Hanna Kavli Lodberg-Holm, Christian Robstad, Theodoros Kominos, Antonia Galanaki, Giannis Gasteratos, Theodoros Naziridis, Richard Gunner, Vaclav Silovsky, Miloš Ježek, Holly English, Olivia Shott, Katie Bambridge, Amy Fuller, Caitlin Willoughby, Elliot Dee, Hazel Nichols, Flavio Quintana, Andreas Fahlman, Josefin Larsson, David M. Scantlebury, Ursula Siebert

**Affiliations:** ^1^ Department of Biosciences, Swansea University, Swansea, UK; ^2^ College of Science, Swansea University, Swansea, UK; ^3^ Department of Biosciences, Swansea University – Singleton Park Campus, Swansea, Wales, UK; ^4^ Department of Biology, University of South-Eastern Norway, Kongsberg, Bø Telemark, Norway; ^5^ Department of Zoology, Aristotle University of Thessaloniki, Thessaloniki, Greece; ^6^ ECOSTUDIES P.C./Environmental Studies, Athens, Greece; ^7^ The Natural Environment and Climate Change Agency, Kerkini Branch, Kerkini, Greece; ^8^ Department for the Ecology of Animal Societies, Max Planck Institute of Animal Behavior, Radolfzell, Germany; ^9^ Department of Game Management and Wildlife Biology, University of Life Sciences, Prague, Czech Republic; ^10^ University College Dublin, Dublin, Ireland; ^11^ Swansea University – Singleton Park Campus, Swansea, Wales, UK; ^12^ Instituto de Biología de Organismos Marinos (IBIOMAR), Consejo Nacional de Investigaciones Cientificas y Tecnicas, Puerto Madryn, Chubut, Argentina; ^13^ Research, Fundación Oceanogràfic de la Comunitat Valenciana, Valencia Area, Spain; ^14^ Kolmården Wildlife Park, Kolmården, Sweden; ^15^ Department of Biological Sciences, Queen's University Belfast, Belfast, UK; ^16^ Institute for Terrestrial and Aquatic Wildlife Research, University of Veterinary Medicine Hannover, Foundation, Büsum, Germany

**Keywords:** bur-tagging, biologging, biotelemetry, animal capture, animal restraint

## Abstract

The use of electronic tags has significantly advanced our understanding of wild animal behaviour and physiology. However, traditional tagging methods often require capturing and restraining or sedating animals, which causes stress and may potentially affect data quality during acclimatization. Inspired by plant burs, we propose a novel ‘bur-tagging’ system to attach tags without capture or restraint. We outline a framework for bur-tagging, detailing the design and key considerations for its success. This includes the influence of tagging site location and animal neophobia on the likelihood of tagging over time, strategies to target specific species, and methods to improve tag placement accuracy. The choice of adhesive mechanism and application force are discussed as critical factors for effective attachment. Preliminary trials highlight animal reactions to inactive tagging systems, demonstrating ways to minimize stress and increase tagging efficiency. Field tests on domestic animals and wild canids in Greece suggest that bur-tagging is a viable alternative to conventional methods. While still in development, bur-tagging has the potential to deploy advanced electronic tags on wild animals with reduced stress and greater ethical consideration, offering a promising tool for wildlife research. This innovative approach bridges biology and technology to address challenges in animal tagging.

## 1. Introduction

The deployment of tags on animals has become a subdiscipline within zoology in its own right (often termed ‘biologging’ or ‘animal biotelemetry’). It has progressed from the use of simple VHF systems in the 1960s [1] to the hugely sophisticated transmission and logging systems used today [2] that can record ‘big’ data simultaneously from multiple sensors, such as accelerometers, magnetometers, pressure and temperature sensors [3], as well as from microphones and cameras [4]. These tags not only provide unprecedented detail on the movements, behaviours and energy expenditures of individual animals [5] in addition to documenting details of the animal’s environment [6] and recording physiological function [7], but are now being used in such numbers that researchers are rapidly moving towards using data from them to examine ecosystem functioning [8] and how physiological limitations may curtail survival and help guide conservation efforts [7,9]. Indeed, the potential of animal-attached tags for understanding fundamental and applied issues in wild animal behavioural ecology [4] and eco-physiology [7,9] is such that thousands of tags are deployed across the globe every year.

However, almost without exception (but see e.g. [10,11] for pole spear tagging of whales and sharks), these tags are placed on (or in) the carrier animals after capturing them [12,13], either by trapping them and/or by using sedatives [14]. This process causes considerable stress, both physiological and behavioural ([14] and references therein), affecting animal well-being and influencing the scientific value of the results [15]. Given that a major prerequisite for deploying tags on wild animals is that it should maximize welfare [14], there is obviously a need to optimize best practice for tag deployments [16]. Although there is much literature on how to do this within the ‘animal capture and restraint’ model of tag deployment [17], we suggest that it should now be possible to deploy tags on to study animals without capture and restraint. Instead, we propose that tag deployment can be effected by an automatic tag dispenser that releases the tag on to the passing animal, at which point the tag should adhere for an undefined period, providing useful data without restraint. In support of this general approach, we note that a large variety of plants disperse seeds—termed burs—using this method [18] so we accordingly call our proposition ‘bur-tagging’. Indeed, given the extraordinary miniaturization of animal-attached tags, the viability of bur-tagging follows as an almost logical consequence.

In this work, we present an initial framework for bur-tagging—an approach designed for furred animals and grounded in interdisciplinary research aimed at quantifying the key factors that influence deployment success. Bur-tagging can be achieved through two fundamental mechanisms: (i) *contact-based systems*, where the animal physically brushes against a fixed tag (as seen in seed dispersal strategies of plant burs and in the ambush tactics of ticks [19]), or (ii) *projected systems*, where a tag is launched on to the animal as it passes a sensor-equipped station. We developed and tested both approaches. Rather than proposing a universal solution, we aim to provide a general framework that can be tailored to the species of interest. We believe that, once adopted and refined, this strategy has the potential to enhance data collection quality while improving animal welfare by eliminating the need for capture or restraint.

## 2. Material and methods

### 2.1. Tag deployment system overview

Our bur-tagging system consisted of: (i) a frame on which the hardware was mounted, (ii) a sensor system to detect when the target animal was correctly positioned, (iii) a controller to relay this signal to an actuator, (iv) an actuator that triggered the tag deployment mechanism, (v) a tag projector, and (vi) a tag with an adhesive interface to attach to the animal. The design allowed us to test both projecting and contact-based systems using the same basic infrastructure. The components and their performance are detailed in §2.2.

### 2.2. Basic design for a bur-tagging system

#### 2.2.1. The support frame

**Figure 1.**
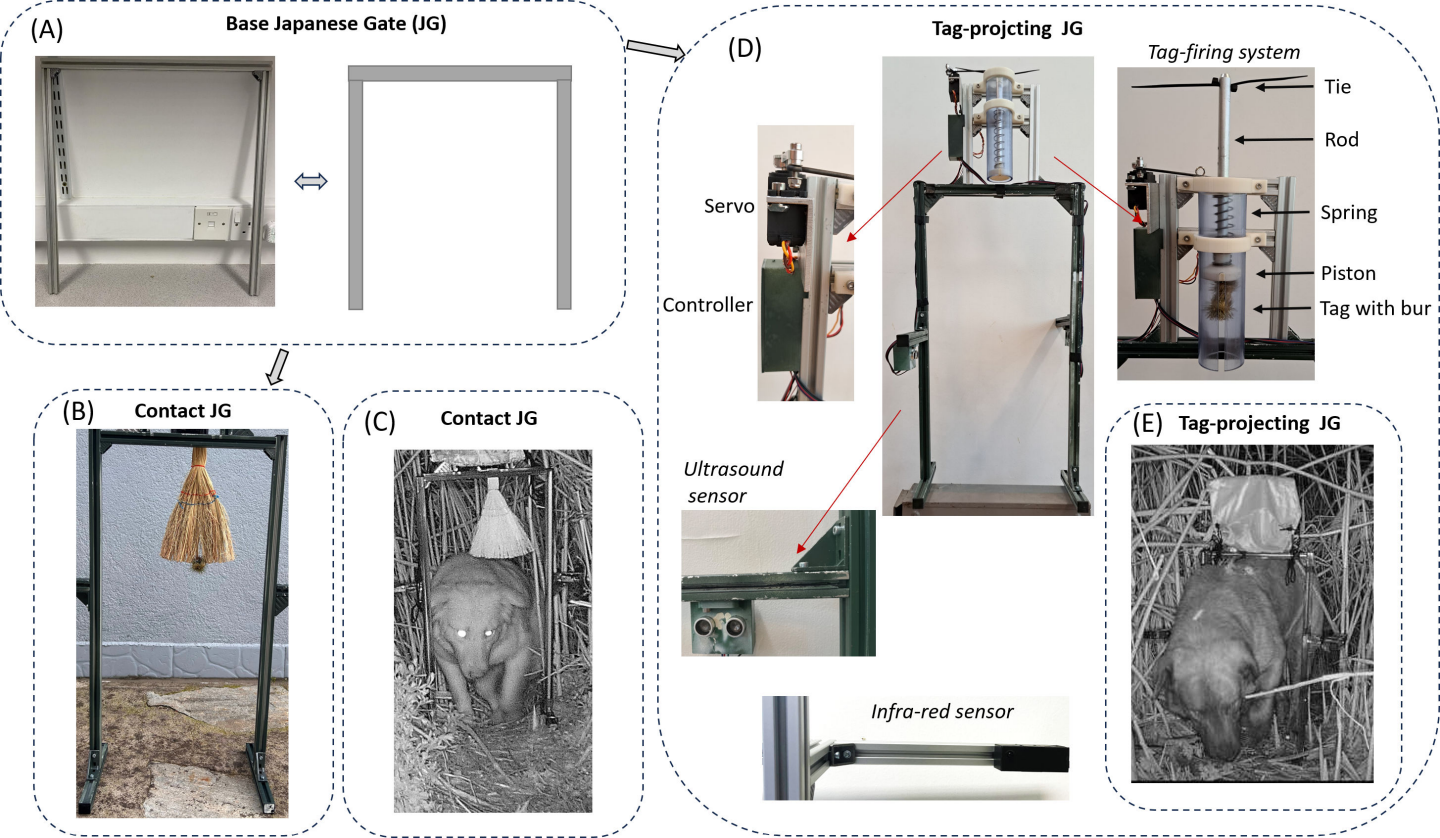
The two bur-tagging systems described in the text. Both were supported by the base Japanese Gate (A). (B) The ‘contact-Japanese Gate’ consisted solely of a brush on which the tag was placed—clearly visible at the base of the brush. (C) shows the set-up in the wild with a feral dog moving through the gate. (D) The ‘tag-projecting Japanese Gate’ consisted of one of two types of sensor (ultrasound—shown attached to the gate here) and infrared, a system controller, a servo and a tag projecting system. (E) Shows a feral dog walking through the Japanese Gate at the point of being tagged where the tag is projected over *ca* 10 cm (for details see text). Here, the tag dispenser has been covered by a bag to reduce its apparent footprint.

Our base frame took the form of an inverted U-shape constructed of profiled aluminium (Bosch Rexroth struts (20 × 20 mm with a 6 mm groove)). The standardized version of our system had both a height and width of 400 mm (figure 1A) although this could be varied easily by sliding the bolts connecting the three sections together. We refer to this base support frame as the ‘Japanese Gate’. For contact between the legs and the substrate, we either used two steel spikes, which could be screwed on to the base of each side strut (for soft substrate) or two T-pieces (for hard substrate).

#### 2.2.2. Contact-Japanese Gate

In the ‘contact’ system, we simulated plant burs by attaching a brush to the horizontal bar, with the fibres projecting down (figure 1B). The tag was placed in the centre line of the brush. Target animals passing through the gate, pushing through the brush, could result in the tag adhering to the animal (figure 1C).

#### 2.2.3. Tag-projecting Japanese Gate

**Figure 2.**
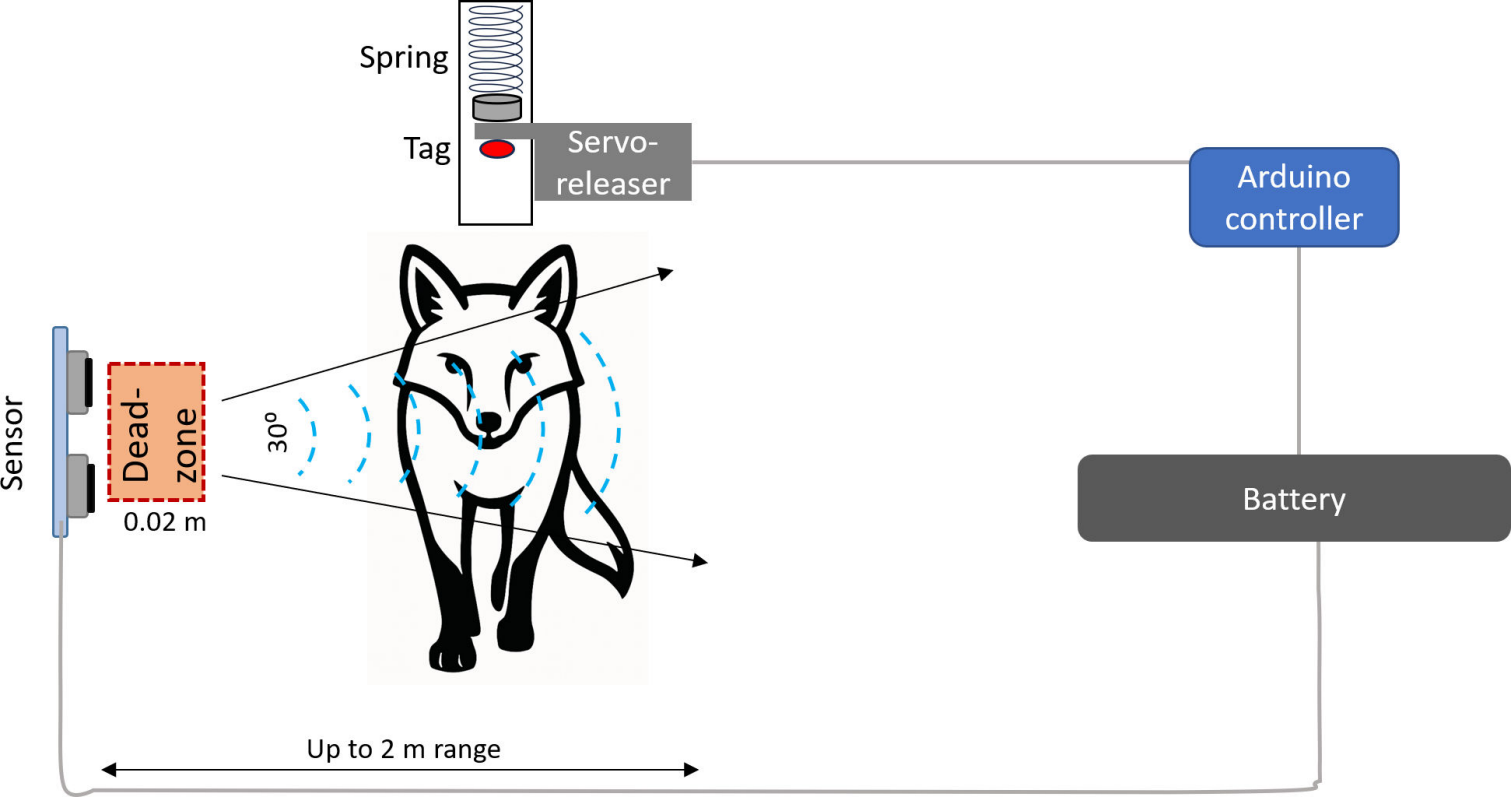
Schematic diagram of the main components of the projecting bur-tagging system using the ultrasound sensor.

Our tag-projecting Japanese Gate incorporated five elements beside the gate itself (figures 1 and 2).

##### 2.2.3.1. Animal sensors

We tested two types of sensor. These were (i) two ultrasound sensors (ELEGOO) or (ii) infrared transmitters and detectors (using an LM393 comparator)—where the blue adjustable resistor set the threshold for the comparator against the incoming detected voltage level. The ultrasound sensors were attached to aluminium arms projecting perpendicularly, but parallel to each other, from the vertical struts of the Japanese Gate, one on either side (figure 1D). The infrared transmitters and receivers were similarly mounted on aluminium arms projecting perpendicularly from the gate, but with two arms from each side of the gate, one holding the transmitter and the other the receiver. The ultrasound sensors worked by transmitting a high-frequency signal and listening for a reflection to indicate that an animal was in front of the sensor [20]. The operational distance over which the ultrasound worked was programmable and, according to maker’s specifications, varied between 0.02 and 2 m.

The infrared system produced a beam of light (angle 11°) that was sensed by the receiver opposite (detection angle 35°). A break in the beam indicated that an animal had moved between transmitter and receiver. The system was developed to work only during darkness for night-active species so that the Japanese Gate could be left ‘operational’ but inactive throughout the day.

##### 2.2.3.2. System controller

The system was controlled by an Arduino Nano microcontroller board, powered by a rechargeable lithium battery (Miady 5000mAh). It monitored either two ultrasound sensors or two infrared sensors equipped with infrared LEDs, which were positioned directly opposite their detectors. These LEDs were activated every 100 ms to check if anything was obstructing the light beam. When both sensors simultaneously failed to detect their respective infrared LEDs, indicating that the animal was correctly positioned between them, the control system triggered the servo motor’s output arm to rotate (electronic supplementary material, S1), releasing the pin that held a spring-loaded actuator in place (see §2.2.3.3).

##### 2.2.3.3. Actuator

We used a servo motor (MMOBIEL SG90) powered by the lithium battery (see §2.2.3.2) to retract the pin which, otherwise, prevented the tag propulsion mechanism (see §2.2.3.4) from being activated. Specifically, when the servo rotated, the pin was retracted from the mechanism blocking the spring-loaded actuator (figure 1D), requiring a movement of only about 2 mm. This ‘hair trigger’ approach was required to minimize the time from servo activation to actuation because servos make a small amount of noise, and it was considered that this might affect the target animal.

##### 2.2.3.4. Tag projector

The tag was ejected from a housing located on the horizontal bar of the Japanese Gate (figure 1D). The driving mechanism was a compression spring placed vertically to accelerate the tag downwards when triggered. We tested two designs; a larger spring (S_L_) (alloy steel compression; 110 × 21.6 mm diameter × 1 mm thickness, 0.99 N mm^−1^) within an acrylic (projecting) cylinder (dimensions 194 × 53 mm diameter)—described in detail here—and a small spring (S_s_) (0.15 N mm^−1^—dimensions 62 × 6 mm diameter) within a rectangular three-dimensional printed housing. The larger spring terminated at its lower end in a piston (40 mm diameter), to the diametric centre of which an aluminium rod (202 mm long × 24 mm diameter) was attached, which ran up the length of the projecting cylinder. The rod had four holes (each of diameter 3 mm) along its length, spaced at a distance of 15 mm. The upper end of the spring was attached to the inside of one end of the projecting cylinder (figure 1D). Pulling the rod upward compressed the spring along the length of the cylinder, bunching it at the upper end. A lever arm attached to the servo motor (see §2.2.3.2) affixed to the gate was articulated with a pin which fitted inside one of the drilled holes and prevented the spring from expanding until the motor was activated and pulled the pin out. When this happened, the spring expanded, accelerating the piston along the length of the projecting cylinder. It was precluded from exiting the cylinder by a plastic tie placed through a further terminal hole in the rod at its uppermost position (figure 1D), which made contact with the upper end of the cylinder. However, when a tag was attached to the piston, it was projected from the cylinder at speed (determined by the extent of compression of the spring before release). The tag was attached to the lower side of the piston by a thin cotton strand, which ran through a small hole in the piston up the length of the cylinder to be attached to the top end of the cylinder when the spring was compressed before tag projection. On projection, the thread broke (figure 1E).

##### 2.2.3.5. Tag adhesion

Tags had adhesive surfaces to stick to the target animals once contact had been made. Systems used for this were; (i) natural burs and (ii) three-dimensional printed simulations of natural burs.

### 2.3. Performance of the bur-tagging system

#### 2.3.4. Sensors—ultrasound

Our ultrasound sensors could be programmed to detect a signal reflected from distances between 0.02 and 2.0 m (maker’s specifications). Since the reflection of sound depends greatly on the reflecting surface, we tested the extent to which the distance between source and sensor depended on animal species fur. For this, the range over which the ultrasound sensors could work and the variability in the ultrasound reflectivity of various animal pelts (dorsal mid-line between the shoulder blades of the pelts) was tested on 15 diverse species of mammal (electronic supplementary material, S2) at the British Museum (a minimum of four runs on five different individuals per species) by setting the standard operating distance of the sensor to 25 cm and approaching the pelt perpendicularly between the shoulder blades from 2 m, noting the distance at which the sensor first detected a reflected signal.

#### 2.3.5. Sensors—infrared

Our infrared animal detection system did not show any distance limitations like the ultrasound sensors for the ranges we worked with. However, we noted any issues with deploying this system, particularly outside the laboratory.

### 2.4. Accuracy of tag placement on the target animal

Our tag projection system was tested for accuracy by projecting a standard test tag (33 × 33 × 33 mm, mass 10 g) from a variety of heights on to an animal pelt (Red deer *Cervus elaphus*). The tag had no adhesive but was dusted with chalk powder so that the point of impact could be determined. We measured the distance of the tag impact site from the centre (directly below the tag projection system) and noted in which of eight compass sectors it fell.

In separate trials, we used our standard test tag and marked one side to represent the side with the adhesive mechanism (see §2.5). This side was loaded facing down within the projection system and placed 39 cm above a fake fur pelt. We filmed the projection process and noted the orientation of the tag at the point of impact with the fur (specifically whether the marked side hit the fur first).

### 2.5. Adhesion between the tag and fur

There are many mechanisms used by plants to ensure that their fruits adhere to carrier animals [21], the two principal ones being hooks or viscid outgrowths (structures that secrete sticky substances). We attempted to simulate the adherence properties of hooks by creating an adhesive pad that we stuck to test tags that consisted of (i) three-dimensional printed simulations of natural burs and (ii) natural burs of the greater burdock *Arctium lappa*.

#### 2.5.1. Adhesion using a standardized applied pressure

**Figure 3.**
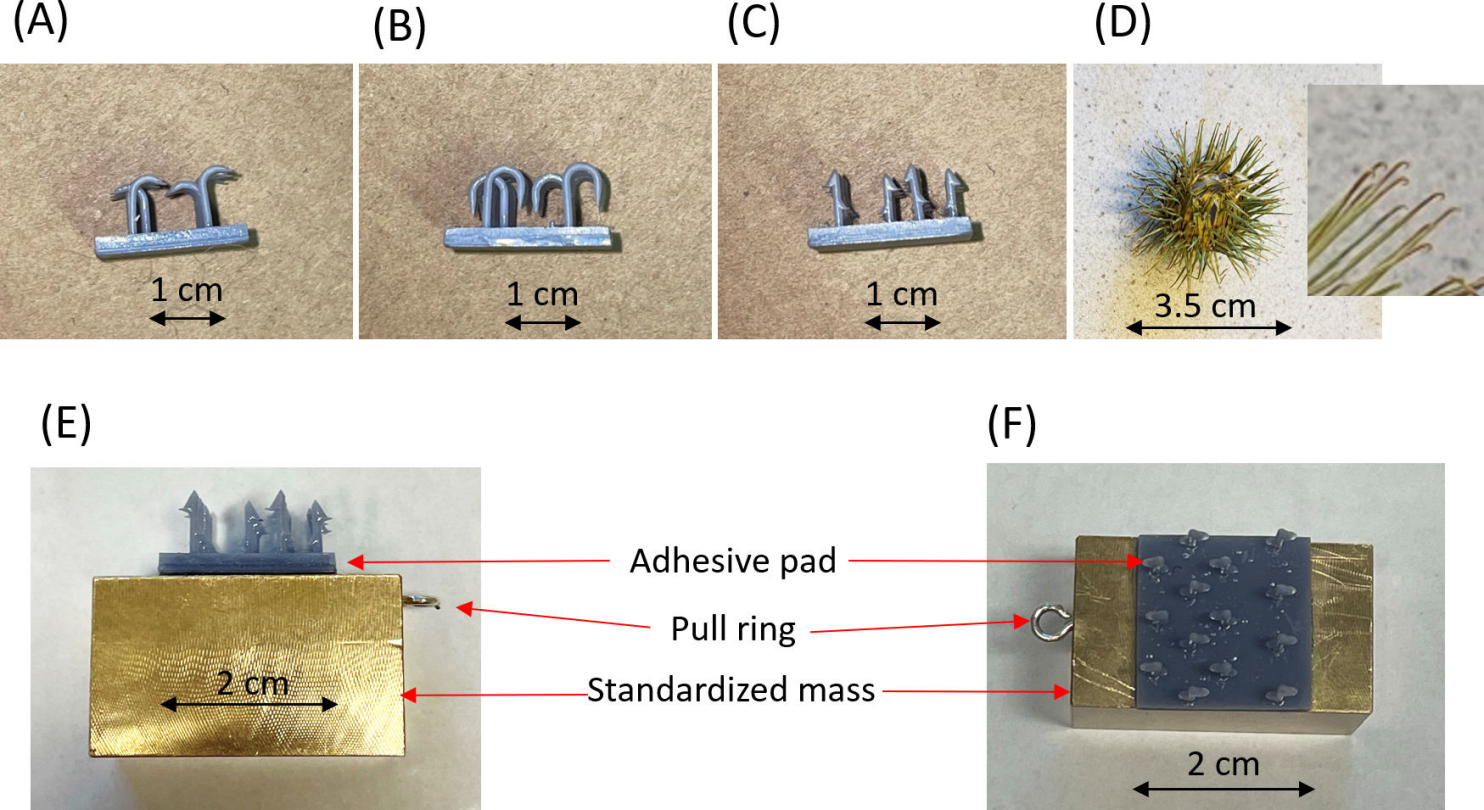
(A–C) Examples of printed simulations of the adhesive structures of natural burs (hooks and barbs) and (D) a bur of greater burdock with inset to show its adhesive hooks. (E) Attachment of one of the adhesive structures (arrow heads) to a standardized mass (test tag) for removal force tests on animal fur. (F) Dorsal view of D showing a pull ring used in the measurement of the force required to remove the tag (see text).

We standardized a tag adhesive surface made of a defined area of plastic (20 × 20 × 0.5 mm), on to one side of which three-dimensional printed hooks and protrusions (all with length standardized to 8 mm) were attached (figure 3A–C). Due to natural variability in the dimensions of greater burdock burs (where the whole infructescence typically measures about 35 mm in diameter, composed of outer hooked involucral bracts and the central capitulum receptacle approximately 15 mm diameter (figure 3D)), it proved impossible to have a standard footprint. Instead, we glued a single bur (figure 3D) to the plastic and used this as our adhesive surface.

The tag adhesive surface was then stuck to a test tag of defined mass (figure 3D,E), which applied a standard vertical force of 3.72 N (applying a pressure of 9300 N m^−2^). The combined effect of adhesive mechanism and standard application force was tested on fake fur by placing the apparatus on the fur before pulling it horizontally with a spring balance and noting the force needed to cause it to move. Unlike in mammals, fake fur has no directionality so pull direction was unimportant as long as it was horizontal. Although we tested a number of different three-dimensional printed designs, we finally standardized all measurements using an arrowhead design with no directionality and 14 arrowheads per adhesive pad (density 35 000 m^−2^) (figure 3D,E).

To examine the extent to which adhesion varied according to potential target species with their various fur types, we repeated the measurements made using the arrowhead test apparatus on 16 mammal species (four measurements made on five individuals per species—species list in electronic supplementary material, table S1). Here, we placed the test apparatus between the shoulder blades of pelts and noted the force required to pull it both (i) in line with the fur and (ii) perpendicular to the fur line using a spring balance.

In tandem, we defined some of the properties of the animal furs used in our tests in an attempt to understand how fur properties might relate to adhesion. For this, we measured hair diameter (between the shoulder blades) at a point half way along the hair length (using a micrometer) and air layer thickness of the fur using a ‘featherometer’ as described in Ainley and Wilson [22] between the shoulder blades of animal pelts. Briefly, the featherometer applies a standard pressure to the fur (applying a perpendicular pressure of 0.5 N cm^−2^) and then measures the distance between the pressure application plate and the skin. Both these measurements were made four times on five individuals from each species.

#### 2.5.2. Tag adhesion to fur following tag projection

**Figure 4.**
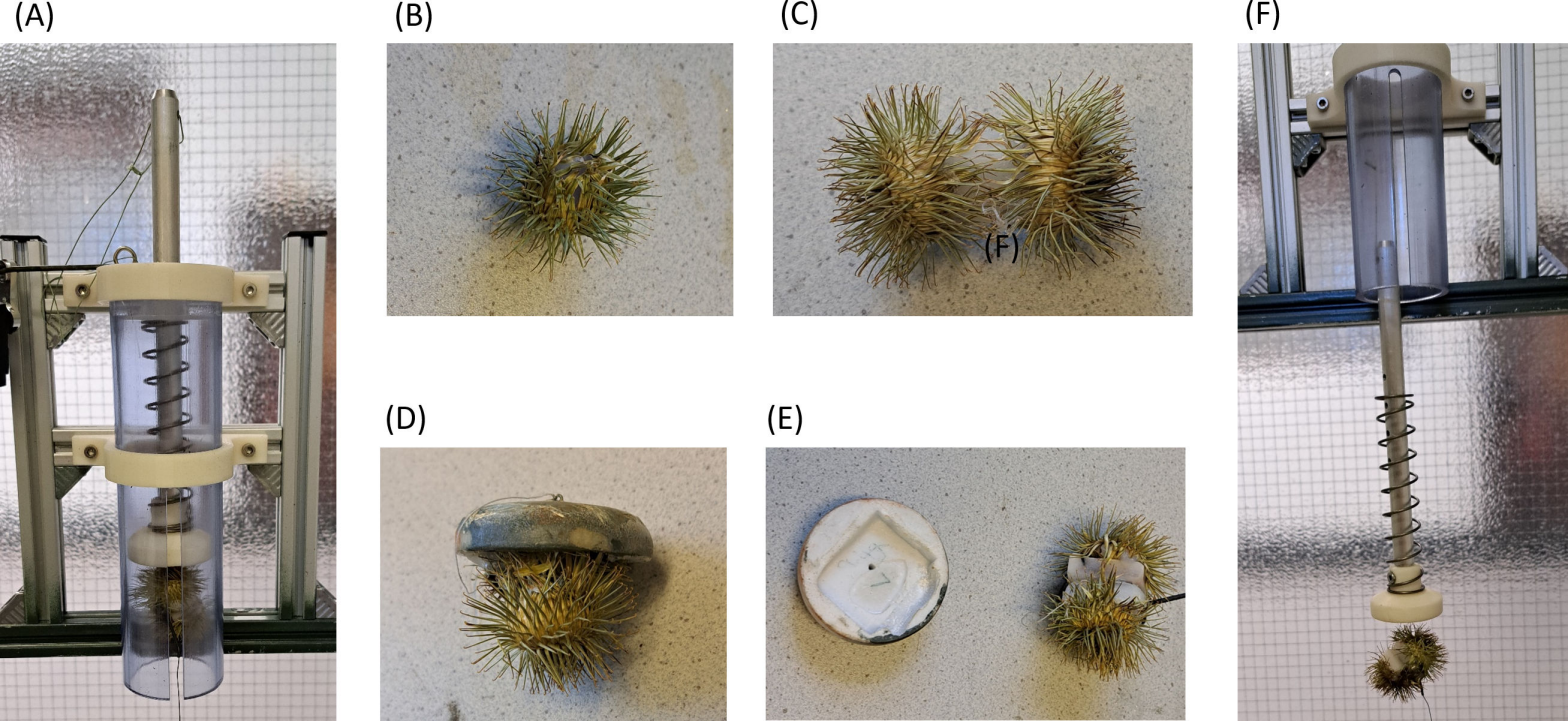
(A) A bur-tagging dispenser loaded with a two-bur tag prior to being armed, (B) a single bur, (C) a tag between two burs, (D) a tag between two burs but fixed to a drive puck, (E) a two-bur tag dissociated from its drive puck, and (F) a two-bur tag being driven by the full mass of the piston.

Initial tag adhesion to animal fur following normal deployment of the bur-tagging system depends more generally on the speeds with which the tag is fired and the forces developed on impact than constant pressure (although constant pressure is a useful approximation for contact-Japanese Gate tagging). Consequently, we examined tag adhesion properties after being fired at the fur by the dispenser. For this, we only used greater burdock burs as adhesives, using S_L_ and being fired vertically down on to European rabbit *Oryctolagus cuniculus* and red deer pelts over a distance of 15 cm (figure 4A). We deployed five bur-tag configurations to explore the role of the amount of adhesion provided by the tag and the mass of the whole system being fired on to the animal. Configurations were (i) a single bur (figure 4B), (ii) a dummy tag (mass 10 g) with two burs (figure 4C), (iii) a dummy tag attached to a ‘drive puck’—a casing intended to increase the mass of the unit being fired (total mass 19 g) using a single bur for adhesion (figure 4D), (iv) a dummy tag associated, but not attached, to a ‘drive puck’ using two burs for adhesion (figure 4E), and (v) a dummy tag attached to two burs driven by the full mass of the drive piston within the tag dispenser (in this case, the usual stop mechanism for the piston (see §2.2.3.4) was removed) (cf. figures 1 and 4F). Here, the total mass of the unit being projected down was 69 g.

Following deployment of the tags on to the fur, we used a spring balance to pull the units both perpendicularly away from the fur as well as horizontally (perpendicular to the line of the fur) noting the force exerted to pull them off.

### 2.6. Reaction of animals to the Japanese Gate

We examined the reaction of domestic dogs *Canis familiaris* to the tag projection system. We primarily used S_S_ although some trials were run on dogs using the S_L_ system (see also preliminary field trials). Dogs (of a variety of breeds) were induced to move through the Japanese Gate using food or encouragement from the owners. At the point the projection system became active, we noted the reaction of the dogs, rating it as 1 (no observable reaction), 2 (mild response = any of: detectable change in behaviour such as a brief pause (less than 1 s), flinching, minor changes in speed but no change in trajectory) or 3 (strong response = any of: extended pause (greater then 1 s), change in trajectory, severe flinching, squatting, change of speed). We also opportunistically observed the reaction of some captive animals (Kolmården Wildlife Park, Sweden) to gain an idea of variability between species. Finally, we filmed the reaction of wild red deer (in the Czech Republic) moving in a group to the tag projection mechanism of the smaller spring system. In this case, there was no gate: We used an ultrasound sensor system that triggered the tag-projecting mechanism from a unit placed at approximately deer head height.

### 2.7. Reaction of wild animals to the base Japanese Gate

We deployed trail cameras at 22 sites in South Wales (electronic supplementary material, S3) over a total of 304 camera days during 2022. The cameras (Wechamp 20MP 1080P HD with SanDisk ultra 32 GB SD cards) were set to record at 1080P for 30 s, with a 40 s lag and high motion sensitivity. Cameras were set to record interactions between any animals and our standardized (non-primed) Japanese Gate (figure 1A), but we considered only European badger *Meles meles* and red fox *Vulpes vulpes* data in any detail as our nominal target animals. Cameras were set in locations that particularly varied according to the visibility of used animal trails, categorized as being: no trail visible, a faint trail or a well-used trail. Due to differences in visibility and topography at the various sites, it was not possible to standardize camera angle or distance to the gate. Gates were nominally left in place recording, unchecked, for a number of days to reduce disturbance but, depending on animal interactions, could be redeployed at the same site. For 199 of these camera days, we used bait (‘Brambles meaty hedgehog food’—https://www.littlepeckers.co.uk/p/brambles-meaty-hedgehog-food—7 g placed below the centre of the gate and 2 × 7 g, one placed either side of the gate at 20 cm) to examine if this would increase the chances of animals interacting with the system.

### 2.8. Preliminary field trials using the projecting bur-tagging system

We tested our tag-projecting Japanese Gates and contact-Japanese Gates on a population of golden jackals *Canis aureus* and feral dogs around Kerkini, Greece during March 2024. We had a total of 25 gate-nights (over 10 days) of gate deployments, of which three gate-nights were non-primed gates and 22 gate-nights were primed with VHFs (ATS Avian Glue-On A2400) using two greater burdock burs (with the seeds removed) as adhesives. Of the primed gates, four gate-nights were allocated to the contact gates and 18 gate-nights to the infrared tag-projecting gates. All bur-tagging systems were filmed using Bushwhacker Camo 4G camera traps, with the data being transmitted live to our base camp. We also used an infrared drone (a DJI Mavic 3T) to examine the behaviours of target animals around the bur-tagging systems. Following deployment of the tags, where possible, we traced the position of the tagged animal (or fallen tag) by triangulating on the VHF signal in the days following deployment.

## 3. Results

### 3.1. Animal detection sensors

#### 3.1.1. Ultrasound sensors

**Figure 5.**
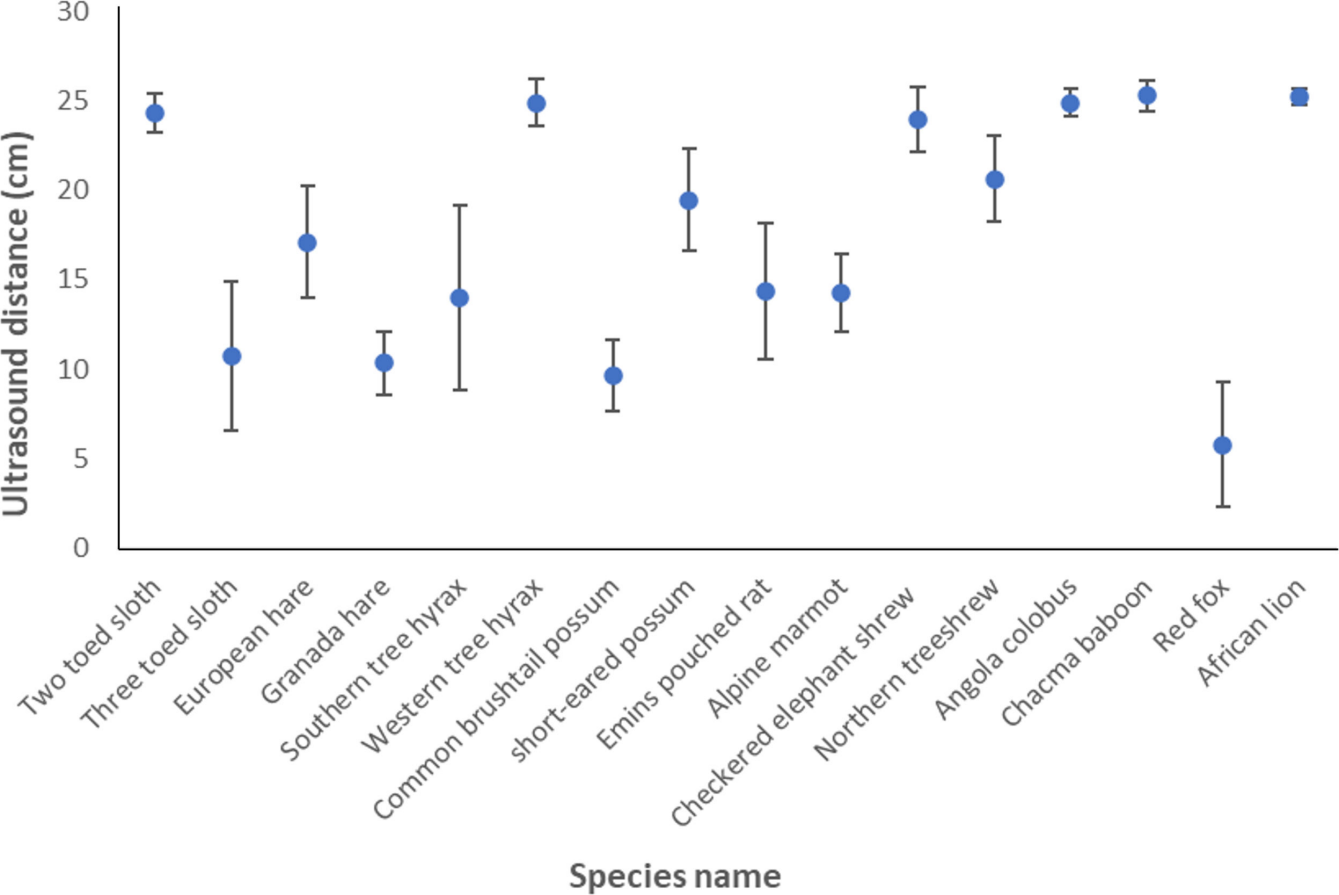
Variation in the distance at which an ultrasound sensor, set to be triggered at a distance of 25 cm, actually triggers according to species (species were represented by pelts and tested in the mid-line of the back between the shoulder blades). Each point represents a grand mean consisting of four measurements per individual across five individuals per species (error bars are s.d.). Latin names for the various species are given in electronic supplementary material, S2.

There was considerable variability in the reflectivity of the ultrasound provided by our systems according to species, with detectable reflected signals varying between mean distances of about 5 and 25 cm (figure 5).

Our ultrasound sensors also seemed to be sensitive to humidity, not functioning in about a quarter of outdoor test trials when humidity was high (greater than 90%). Although we attempted to quantify this in the laboratory, we could not identify any obvious general patterns.

#### 3.1.2. Infrared

While the infrared emitters and sensors performed reliably in laboratory tests and were not subject to the same reflection problems as ultrasound sensors, setting them up outdoors on uneven ground proved challenging. The slight ‘play’ in the Japanese Gate mounting could cause the narrow 11° beam from the emitter to miss the sensor. However, because the beam could be seen through a mobile phone camera, minor adjustments were straightforward to make.

### 3.2. Tag dispenser properties

**Figure 6.**
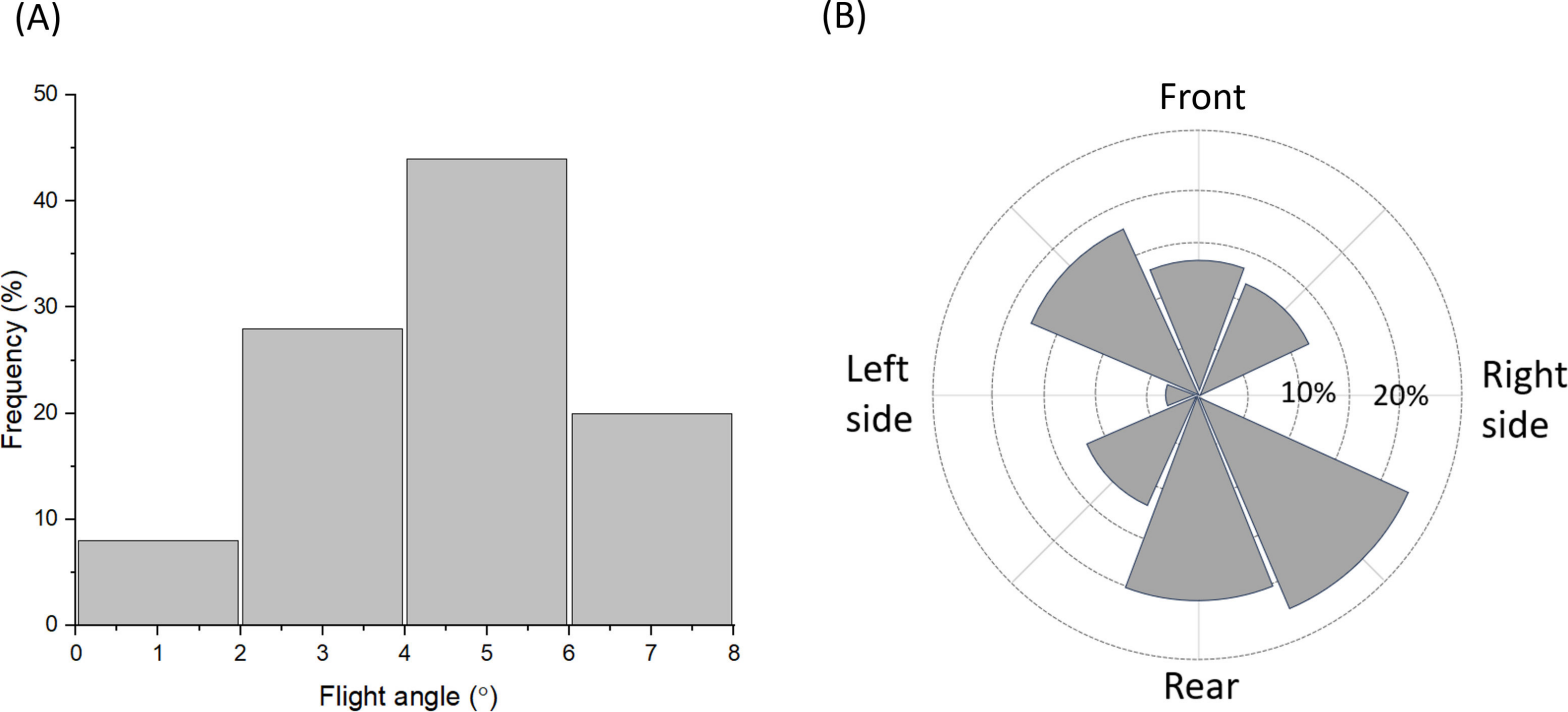
(A) Flight angle and (B) sector occupation of 7.4 g tags being projected vertically down over 146 cm (*n* = 50). Note that flight angle does not have a mean error of 0° and the fall sector relative to the dispenser is not even.

Tags powered down by the spring system deviated appreciably from a straight-line trajectory. For example, in 50 trials where a tag (20 × 20 × 10 mm, mass 7.4 g) was fired from the S_S_ system at a pelt 146 cm below it (a much greater distance than we would normally operate for animals but useful to indicate limits), mean angular deviation from the vertical was 4.6° (s.d. 1.8) with no mode around zero (figure 6A) and an uneven radial distribution of points (figure 6B).

Orientation of tags was not maintained during flight. During trials where tags (33 × 33 × 10 mm, mass 12.5 g) were fired 39 cm vertically down at a test pelt using the S_S_ spring system, 31.1% flipped their initial projection orientation ($\chi^{2}$ = 14.71, *p* < 0.0002, d.f. = 1, *n* = 210 trials). This would obviously affect the probability of adhesion if the tag had only one adhesive surface.

### 3.3. Adhesion of the tag to fur

#### 3.3.1. Tag adhesion to fur under constant pressure

The adhesion of our standardized tag to animal fur, as measured by the force to move the tag in a given direction, depended on the adhesive surface and the fur. For example, the three different adhesive structures shown in figure 3A–C required 78.5 mN (s.d. 48.3), 150 mN (s.d. 140.5) and 32.6 mN (s.d. 60.5) (*n* = 15 in all cases) to be pulled perpendicularly across red deer fur. The corresponding figures for fake fur were 0 mN, 150.4 mN (s.d. 132.9) and 6.5 mN (s.d. 25.3). The variance was high in all our three-dimensional printed adhesive surfaces. These printed adhesive surfaces provided strikingly less adhesion to fur than great burdock, which required 7181.8 mN (s.d. 750) and 4363.6 mN (s.d. 1206) for fake fur and red deer fur, respectively. This is about two orders of magnitude greater than the printed surfaces.

**Figure 7.**
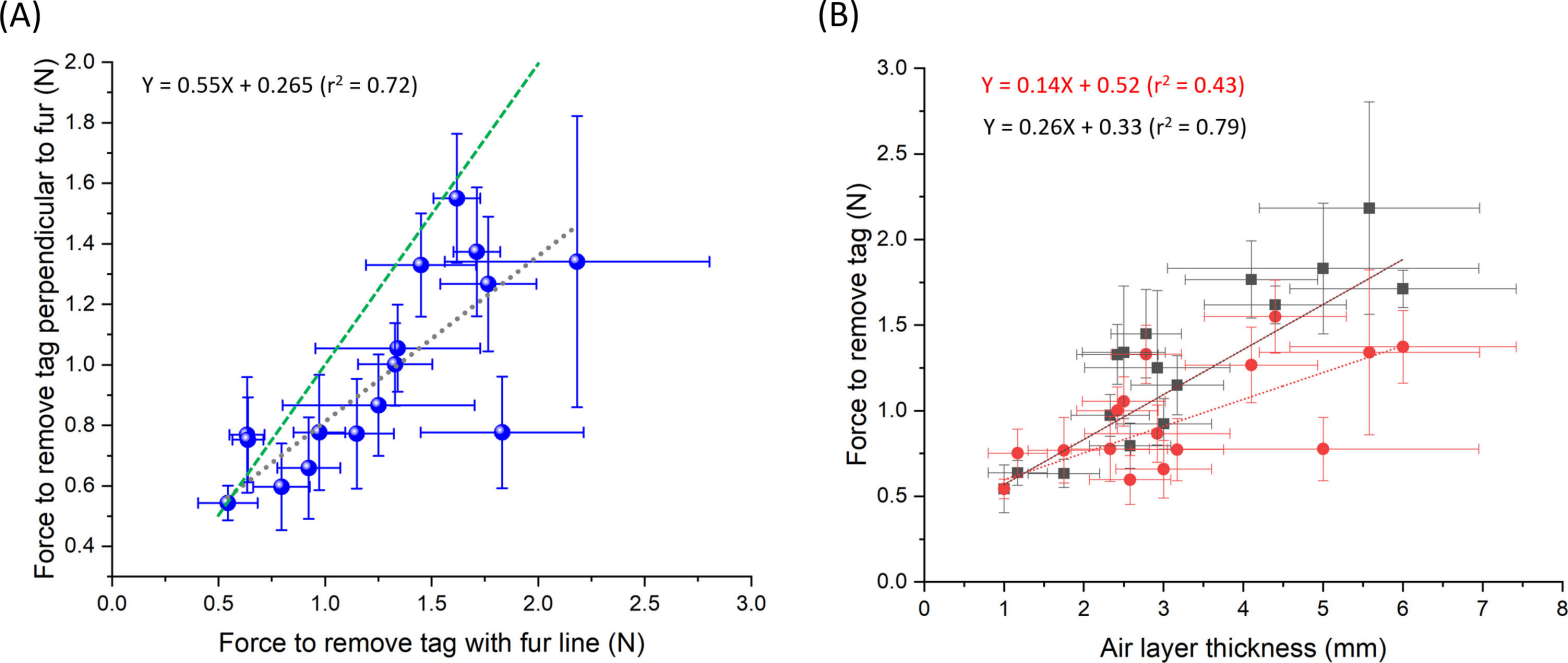
(A) Relationship between the forces required to dislodge a standardized adhesive pad (see figure 3E,F) by pulling in one direction (with the direction of the fur) with respect to the forces required to remove it by pulling in another direction (perpendicular to the fur direction) for 16 different mammal species (see electronic supplementary material, S2 for list—each point represents the grand mean of four measurements made from five individuals per species). The green line shows the line of equality. (B) Relationship between the forces required to dislodge the standardized adhesive pad (A) and species fur thickness (assessed using a ‘featherometer’ applying a perpendicular pressure of 0.5 N cm^−2^—see text) for pull directions ‘with the fur’ (black squares) and ‘perpendicular to the fur’ (red circles) for 16 different mammal species. Error bars are s.d.

The forces needed to dislodge our synthetic arrowhead adhesive pads depended on the direction that the pull force was applied (with the fur, against the fur, or perpendicular to the fur), with higher dislodgement forces in a given pull direction tending to be mirrored by higher forces in another direction (figure 7A). These dislodgement forces also varied greatly with species (figure 7B,C) which was partly explained by a positive relationship with the air layer thickness of the fur (figure 7B).

**Figure 8.**
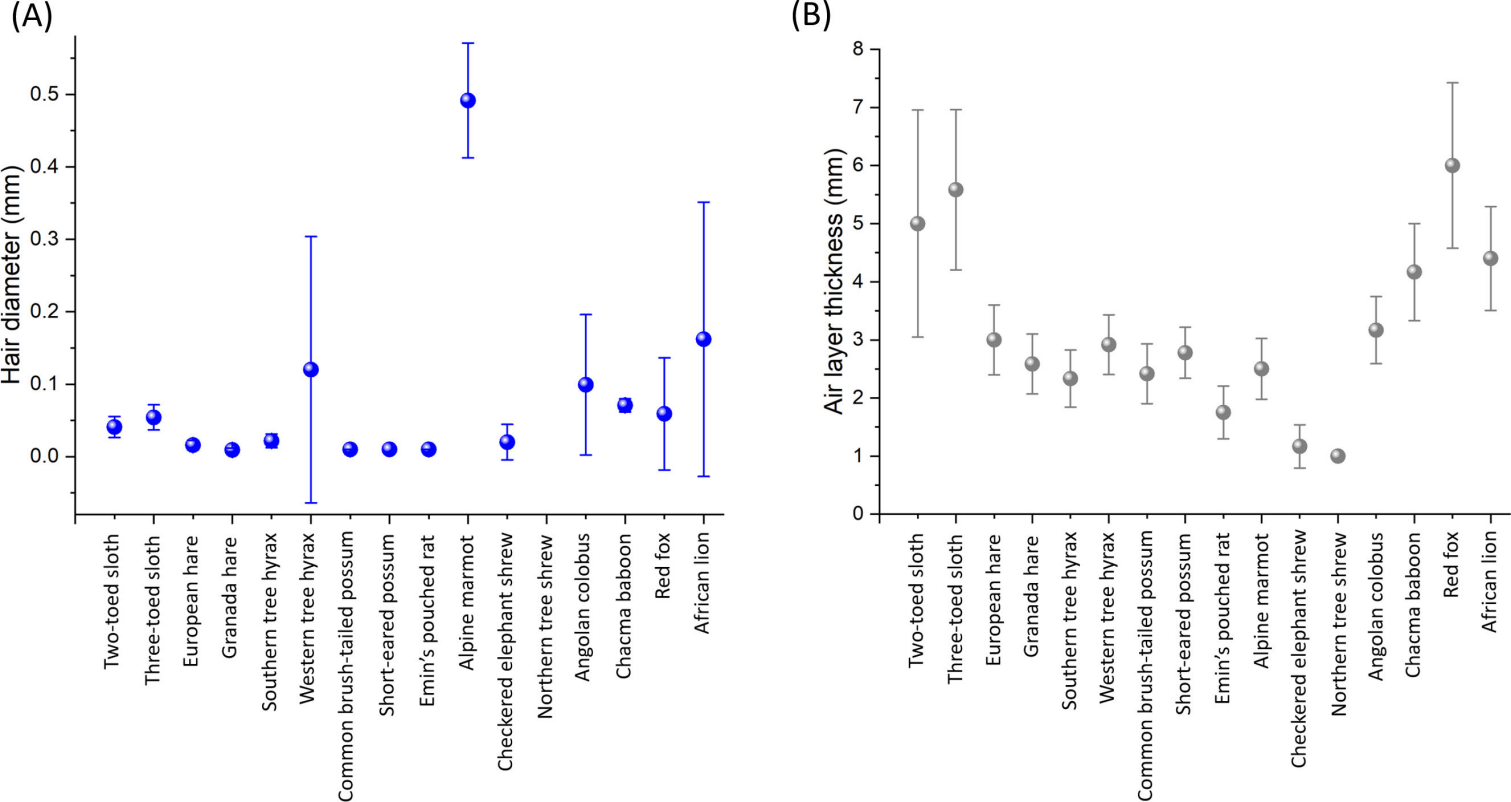
Interspecific variation in (A) fur hair diameter (taken at a point halfway along the hair) and (B) air layer thickness for 16 species of mammal from eight orders (data taken from animal pelts and derived from measurements made in the dorsal mid-line of the back, between the shoulder blades). Each point (±s.d.) is the grand mean from four measurements taken from each of five individuals of that species (see text).

There was no relationship between these forces and fur hair diameter for two of three pull directions (linear mixed effects model for each pull direction; pull ‘with’ fur direction, estimate = 55.7 (s.e. = 59.2), *p* = 0.35, pull ‘against’ fur direction, estimate = 50.0 (s.e. = 45.1), *p* = 0.27, pull ‘perpendicular’ to fur direction, estimate = 76.2 (s.e. = 32.4), *p* = 0.019). Both fur hair diameter and fur air layer thickness showed extensive variation between species (figure 8A,B).

#### 3.3.2. Tag adhesion after projection

**Figure 9.**
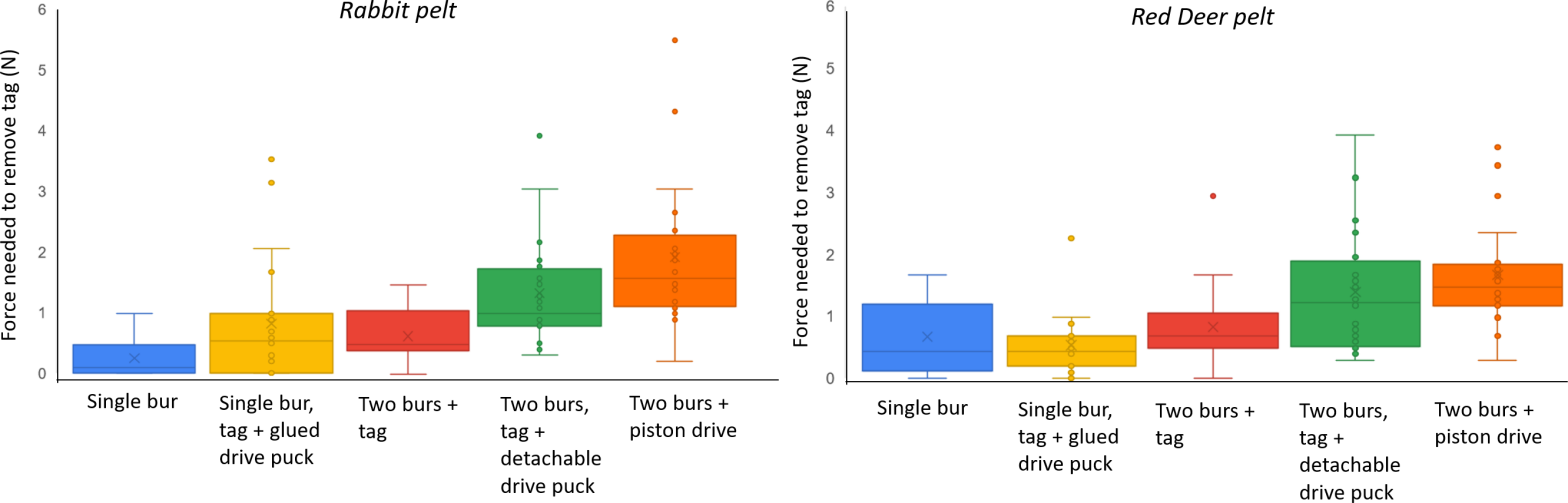
Box whisker plot of the force needed to remove tags (using a perpendicular pull) from rabbit and red deer fur following projection using the tag dispenser (S_L_) shown in figures 1 and 2. Tags were attached to either one or two burs and were given momentum by added masses of either a ‘drive puck’ (see figure 4D,E) or the drive piston (figure 4F). Boxes show the median and inter-quartile range between the first and third quartiles.

Adhesion to fur in tags following projection from the dispenser may not be directly comparable with adhesion from tags applied using defined pressure (see §§2.5.1 and 2.5.2). Tag adhesion following projection varied with removal direction (*p* = 0.018) and increased both with number of adhesive burs (*p* = 0.0046) and the mass of the driver (i.e. the mass of the tag combined with a puck, if used, and/or the drive piston) (*p* < 0.0001) (multi-way ANOVA using: removal force = push force (mass of the tag plus associated puck or piston) + number of burs + animal type + removal direction + interactions) (electronic supplementary material, S4) (figure 9). Multiple regression analysis indicated that the number of burs increased removal force by 379 mN when moving from one bur to two (*p* = 0.006) and that, for each gram of driver mass, the removal force increased by 13 mN (*p* < 0.001). The model explained 22.2% of the variance (electronic supplementary material, S5).

### 3.4. Reaction of wild animals to the Japanese Gate

During deployment of our Japanese Gate, multiple species interacted with it. For example, during 304 days of deployment of the gate at 22 sites in Wales, our camera traps indicated that there were 194 interactions with species from six different vertebrate families (birds, carnivores, mustelids, squirrels, rodents and hedgehogs, in addition to domestic animals). Aside from our target animals (badgers and foxes), a large variety of mammals interacted with the gate, including Eurasian otters *Lutra lutra*, European hedgehogs *Erinaceus europaeus*, brown rats *Rattus norvegicus*, grey squirrels *Sciurus carolinensis*, domestic cats *Felis catus* and domestic dogs *Canis lupus familiaris*. Interactions ranged from the animals simply looking at the system (29% of interactions), pausing (30% of interactions) and sniffing it (41% of interactions). Overall, about 30% of animals appeared attracted to the Japanese Gate while 20% appeared repelled (the animal looked or sniffed toward the Japanese Gate before freezing and backing away or the animal flinched when it first appeared to perceive the Japenese Gate before reversing out of the field of view). Finally, 74% of the individuals walked through the gate while 26% did not.

There was substantial variation in the rate of visitation of animals and the Japanese Gate, with the minimum number of visits for either of our target species being over a 7 days period (e.g. Site 18 for badgers) and the maximum being 19 (badgers) over 11 days (Site 3). We could not generally distinguish with certainty whether one individual visited multiple times or whether such visits were due to multiple individuals.

Site-based sample sizes were too variable and generally too small for detailed examination of changes in habituation or neophobia (the fear of, or aversion to, unfamiliar things [23]). However, we grouped observations of each site into total ‘early’ or ‘late’ numbers of passages through the gate (using the half-way point in deployment time to define the split) before summing them across all sites for a broad-brush view. This indicated that there was no statistically significant difference in the proportion of badgers passing through the gate between the early and late periods (early period—27 of 36 badgers passed through the gate, late period—30 of 35 did so; $X^{2}$ = 0.70, *p* = 0.40, d.f. = 1). It is notable though, that a high percentage (80%) of badgers passed through the gate anyway. Overall, 67% of the foxes passed through the gate, with a higher proportion of foxes passing through the gate in the late period (early period—43 of 74 foxes passed through the gate, late period—51 of 66 did so; $X^{2}$ = 4.97, *p* = 0.026, d.f. = 1).

There was interspecific variation in association of species with trail visibility. Badgers were more often associated with ‘well-defined trails’ than sites where no trail was visible ($X^{2}$ = 6.37, *p* = 0.02, d.f. = 1) although foxes showed no significant difference in their usage of ‘faint’ versus ‘well-defined’ trails ($X^{2}$ = 0.12, *p* > 0.05, d.f. = 1). Finally, baited Japanese Gates significantly increased the frequency of some animal interactions [24] with, for example, there being a highly significant increase in the number of foxes moving through the gate when it was associated with baiting (Poisson test; *Z* = −7.14, *p* < 0.0001).

### 3.5. Reaction of animals to tag deployment

Preliminary experiments with 10 breeds of domestic dogs *Canis lupus familiaris* during a total of 88 passes through the Japanese Gate showed that eight breeds consistently passed with no discernible reaction to being tagged (using the S_S_ system) while the remaining two breeds had no discernible reaction for 94% and 83% of passes but had a mild ‘reaction’ (either a pause of less than 1 s, flinching or minor change in speed) for 6% and 13% of passes, and one of these breeds showed a ‘strong’ response (an extended pause, a change in trajectory, severe flinching, squatting or a radical change in speed) during 3% of passes.

Opportunistic bur-tag deployments on a variety of captive animals (with size ranges from capybaras *Hydrochoerus hydrochaeris* to Bactrian camels *Camelus bactrianus*) showed reactions ranging from no discernible reaction to mild responses. We noted, in capybaras at least, that animals were more hesitant about passing under lower bars (cf. electronic supplementary material, films S1 and S2). Film of the wild red deer travelling in a group showed that a strong startle reaction [25] by one individual to the noise of the gate projecting mechanism induced a similar reaction from adjacent conspecifics (electronic supplementary material, film S3).

### 3.6. Preliminary field trials

Overall, we observed no interaction between canids and the systems over five gate-nights for systems that were primed or otherwise. We never had a jackal move through the gate although there were occasions when large numbers of them surrounded the gates. We had three occasions when feral dogs moved through the gates but were not tagged for technical reasons, either because the power source failed or because the tag was not fully released from the gate (e.g. electronic supplementary material, film S4). Tags were deployed on the dogs on four occasions, three times from the infrared-spring system and once from the contact-Japanese Gate. Of the tagged animals, two VHF tags (tagged by the infrared-spring system) could be retrieved having fallen from the carrier at distances of up to 5 m from the bur-tagging system. One of these had an appreciable tuft of dog hair on it, indicating that it had been pulled off by the animal. However, one feral dog tagged using the infrared-based Japanese Gate was located carrying a tag that appeared very well embedded in the fur *ca* 12 h after deployment 300 m away from the site. This tag was not retrieved and was not relocated on following days. A further feral dog tagged using the contact-Japanese Gate could not be located *ca* 12 h or later after tagging. Since the VHFs had a range of about 500 m, we assumed that the animal had travelled at least this far away from the gate (assuming that the dog had not pulled off the device and broken it in the process).

## 4. Discussion

This work introduces a basic system for tagging some mammals without capture or restraint, focusing on species that pass through a Japanese Gate. While currently limited, the system could be adapted for other species, such as deer, by placing sensors along trails with tag dispensers positioned above, and carnivores, even those that might not obviously follow trails, such as lions, by using mobile tag dispensers that could be driven by remote control to proximity before tag dispensing. We aim to inspire further development of this framework and hope the pointers and mechanisms outlined here will guide future research.

### 4.1. Sensors

#### 4.1.1. Ultrasound

Although our ultrasound sensors generally performed well, humidity posed a significant challenge. These sensors operate by emitting high-frequency sounds and detecting the reflected waves. However, the speed of sound in air is affected by water vapour, with higher humidity increasing the speed and potentially introducing errors in distance measurement. High humidity also attenuates ultrasound waves and may scatter them, reducing signal strength and causing the sensor to miss weak or distant echoes. Additionally, in very humid conditions, condensation can form on the sensor surface or nearby objects, further scattering or absorbing ultrasound waves and degrading sensor accuracy. To address these issues, we recommend using advanced ultrasound sensors that incorporate humidity sensors or algorithms to adjust the speed of sound based on current humidity levels, improving accuracy. Such sensors are typically less than double the price of the units we used and are only a fraction of the total system cost.

Ultrasound sensors allow users to specify their operating distance, enabling precise triggering of the tag-projecting system when the animal is in the correct position. However, the high variability in fur reflectivity to ultrasound waves across species (figure 5) makes it advisable for users to test the sensors on pelts of their study animals to ensure optimal performance.

#### 4.1.2. Infrared

There is a large number of infrared sensors that can be used in the bur-tagging system and we tested just two. We noted difficulties with alignment of emitter and sensor due to the restricted emitter angle, compounded by us not being able to see the beam directly. Using emitters with wider beams will mitigate this but will also use more power, necessitating a larger battery pack to power the system. Our system was also only set to work at night so other sensors should be investigated for daylight tagging.

In this regard, as this project ended, we briefly tested a ‘Time of Flight’ (ToF) laser-ranging module from STMicroelectronics, the VL53L0X. This system has a surface emitting laser (940 nm—invisible to the human eye) and picks up the reflection for distances of up to 2 m (in a similar manner to the ultrasound). We had 10 dogs of various breeds and fur constellations pass through the gate under highly variable light and humidity conditions and it registered 100% of passes. As such, we would recommend this for extensive further testing, nominally as a system of choice.

We emphasize that our bur-tagging system is a modular set-up, with components that can be adapted or replaced to suit specific circumstances. Depending on the target species and environmental conditions, alternative sensor systems may perform more effectively. These alternatives include trip-wires, pressure-sensitive plates, passive infrared sensors, microwave sensors and cameras (both optical and thermal) integrated with AI. This flexibility allows the system to be tailored for optimal performance in diverse scenarios.

#### 4.1.3. Correct placement of the tag on the animal

**Figure 10.**
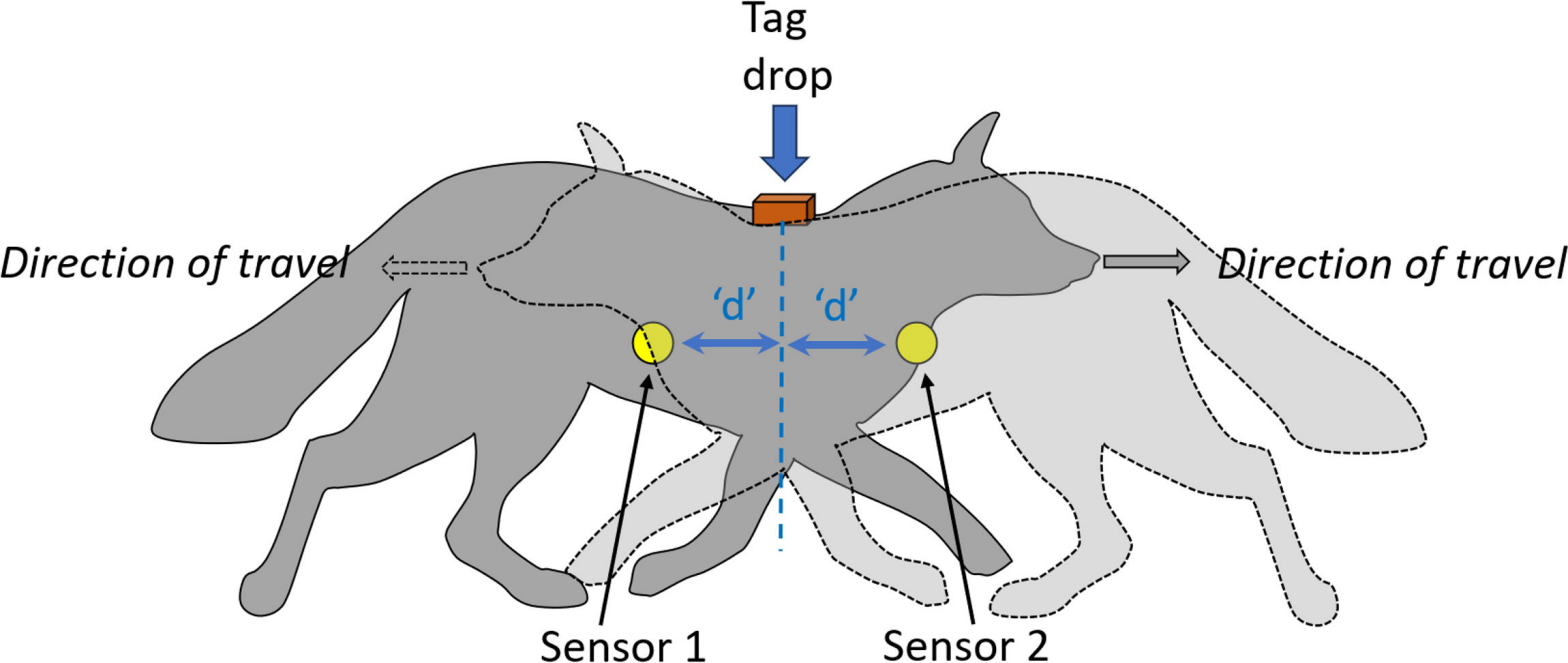
A schematic diagram of a bur-tagging system set-up illustrating a system that allows for bi-directionality of the study animal. The two sensors (yellow circles) should be equidistant from the ideal tag spot and that distance should be equal to the distance between the first point that the animal triggers one of the sensors and the ideal tagging position.

Our bur-tagging system used two sensors to accurately determine the optimal tag placement on the animal, noting that the best body positions to attach biologgers on wild animals is an important subject field in itself (e.g. [26]). Using at least two sensors is crucial for identifying the animal’s direction of travel; without them, direction cannot be determined. The placement of the tag is influenced by the relative distances between the sensors and the tag dispenser. Specifically, the distances were configured to correspond to the ideal tagging location on the animal. This set-up ensures that the tag is deployed at the correct position as the animal passes through the bur-tagging system (figure 10).

We propose that the ideal tagging location on the target animal is between the shoulder blades. This site is not only difficult for the animal to groom but also serves as an excellent approximation of the body’s centre, which is beneficial for tags that incorporate accelerometers [27]. Considering this, our tag dispenser was typically positioned above the animal’s centre line, aligning with the expected trajectory as the animal passes through the Japanese Gate. It is unclear how bur-tagged animals might react if the tag were dropped in a non-ideal spot on their body because biologgers on terrestrial mammals are currently all restrained to be equipped for precise positioning [28]. We note though, that most studies on terrestrial animals equipped with external tag technology use collars [29] although ear tags are also used and at least one study has glued tags to fur [30]. The use of collars around the neck is a convenience that usually ensures long deployment periods rather than being an attachment mechanism that minimizes animal detriment [31]. Examining the reaction of bur-tagged animals to tagging at various body sites will be an important consideration in future work.

We used two sensors to detect target animals, which also play a critical role in avoiding the tagging of non-target species. Adding more sensors can further refine this process. In our standard bur-tagging system (figure 1), the software deployed the tag when both ultrasound sensors were triggered. The height of the sensors above the ground and their detection cones partly account for the length and height of the target species, requiring careful calibration but not entirely excluding similarly sized species. Using multiple sensors at different vertical levels could better define the side dimensions of target animals. In this regard, although sexual dimorphism [32] could potentially be a concern in some species, even pronounced mass differences—such as in polar bears *Ursus maritimus*, where males weigh up to twice as much as females—translate to much smaller differences in linear dimensions, due to mass scaling with the cube of length. For example, male polar bears are only about 16% longer than females [33], and shoulder height is expected to differ similarly. Finally, we suggest that an enhanced bur-tagging system could incorporate AI-linked cameras to precisely determine when the target animal is in the optimal tagging position and to distinguish between species [34]. We expect this avenue to be particularly fruitful in the future [35].

### 4.2. Tag dispenser

#### 4.2.1. Tag trajectory time

Both the trajectory of the released tag and the flight time are critically important to ensure the tag lands in the correct position on the animal’s body (see §4.2.2). The speed of the trajectory directly affects the time taken, and if the animal is moving, slower speeds can result in the tag landing further back on the body. In early experiments, we explored a simple passive drop for tags. However, unless the trajectory distance is minimal, this method was too slow because the tag had to accelerate from rest using only gravity. This contrasts with the contact-Japanese Gate method, where the animal rubs the tag off, resulting in a trajectory distance of zero (see §4.2.2).

We chose compression springs as a tag-projecting force because they provide standardized forces (expressed as N m^−1^ compression—the ‘spring constant’) [36] that can easily be calculated based on how much the spring is compressed. While we recommend testing various springs in the laboratory to evaluate their ability to propel the tag, there is a clear mechanistic link between the choice of spring and the speed of the powered tag, which can help guide selection. Both of these can be used to calculate the tag trajectory time (electronic supplementary material, S6).

Springs with high spring constants increase the speed with which the tag moves between the dispenser and the animal, and, because the speeds are higher, it helps the tag to adhere to the animal on contact (see §4.2.2). Against this, higher compression force springs require a larger housing (increasing the Japanese Gate footprint) and also produce more noise on release, which is more likely to startle the target animal.

#### 4.2.2. Effect of tag trajectory speed and length on optimized tag positioning

Tag speed at release coupled with tag trajectory distance and animal speed can be used to calculate how far from the ideal tagging spot on the animal the tag will fall (assuming no trajectory errors—see §4.2.3). If, for simplicity, we assume a constant tag speed from the release point to contact, the time taken for the tag flight T_flight_ is


(5.1) $${\text{T}}_{\text{flight}}={\text{D}}_{\text{trajectory}}/{\text{S}}_{\text{tag}},$$

where D_trajectory_ is the vertical trajectory distance of the tag and S_tag_ is the tag speed. During this time, the animal will have moved underneath the Japanese Gate by a distance of


(5.2) $${\text{D}}_{\text{animal}}={\text{S}}_{\text{animal}}\times {\text{T}}_{\text{flight}},$$

where S_animal_ is the speed of the animal. Substituting in for T_flight_, the distance down the animal’s back that the tag will fall is


(5.3) $${\text{D}}_{\text{animal}}={\text{S}}_{\text{animal}}\times {\text{D}}_{\text{trajectory}}/{\text{S}}_{\text{tag}}.$$

For example, if the flight time of the tag lasts 0.35 s, an animal travelling at 0.1 m s^−1^ with its shoulders at a distance of 0.1 m below the tag release spot will have the tag landing some 38 mm behind the ideal spot. This increases to 520 mm if the animal is travelling at 1 m s^−1^ and the distance between the animal and tag release spot is 0.5 m. This illustrates the importance of performing such calculations to inform likely outcome and practice.

#### 4.2.3. Errors in tag trajectory

There is an extensive body of literature on projectile trajectory. For our purposes, we note that while compression springs apply a clean, unidirectional force to a tag moving down a barrel—minimizing trajectory errors—several factors, such as a non-streamlined tag shape, can still cause deviations from a perfect vertical trajectory. In our system, mean angle errors were approximately 4−5°, although these errors were not evenly distributed around a central point (e.g. figure 6).

Defining trajectory errors in terms of angle, which can be assessed in the laboratory, is crucial. Errors in tag landing position relative to the ideal position on the animal can be calculated using simple trigonometry based on the tag flight length. The mean error distance, D_error_, from the spot directly below the tag is given by


(5.4) $${\text{D}}_{\text{error}}={\text{D}}_{\text{trajectory}}\;\sin\Theta ,$$

where Θ is the angle error. For example, with an angular error of 5°, a tag moving down a trajectory of 100 mm will land just 9 mm from the ideal spot. However, this error increases to 43 mm if the tag travels 500 mm vertically. Whether these or other trajectory errors are problematic depends on the size of the target animal.

#### 4.2.4. Tag rotation

Our work demonstrated that at a tag trajectory distance of 39 cm, approximately 30% of tags altered their orientation to land with the leading face away from the animal. Tag rotation is influenced by a complex interplay of tag shape and weighting, highlighting the importance of conducting tests to evaluate this phenomenon. In the Japanese Gate constellation that we used for domestic dogs, where tag trajectory was around 10 cm before it reached the target animal, we consider tag rotation to be negligible. However, some target species may require much greater tag flight distances, which are likely to make tag rotation correspondingly more relevant. This reinforces the need to minimize this distance. For cases where trajectory distances are significant, researchers should consider providing adhesion on all sides of the tag or designing a tag shape that ensures stable flight.

### 4.3. Adhesion of the tag to fur

The effectiveness of the bur-tagging method hinges on the tag’s ability to adhere securely to the animal’s fur [37]. This can be evaluated by measuring the force required to detach the tag (figure 7). Our findings indicate that the adhesion strength of the tag is influenced by both the attachment mechanism used by the bur tag (figure 9) [21] and the characteristics of the animal’s fur (figures 7 and 9) [38,39]. We propose that quantifying the forces needed to remove tags from pelts provides valuable insight into the potential for tag loss in the field. Specifically, precise measurements of these forces can help predict whether typical animal movements might dislodge the tags (cf. [37]). For instance, the force generated by the tag during movement is a function of its mass and the acceleration of the animal, as described by Newton’s Second Law (*F = ma*). Studies using accelerometers have shown substantial variation in acceleration across species, ranging from 1 to 7*g* [31]. Therefore, a 10 g tag could generate a (removal) force of up to 687 mN, a 20 g tag twice that. If this exceeds the measured removal force, the tag may be at risk of detachment. In addition, the environmental conditions at the time of attachment are likely to be important, i.e. if the fur is wet (which we did not test), as well as the propensity of the target animal to groom the tag off [40] and the site of attachment (which affects both the properties of the fur and the motivation of the animal to groom it off).

To enhance tag adhesion to animals, researchers should consider both the adhesive surface and the force pressing the tag into the fur. A key factor is the use of a ‘drive puck’ of defined mass to push the tag effectively into the fur (figure 9). The driving force can be calculated as


(5.5) $$F=(0.5mv^{2})/d,$$

where *m* is the mass of the tag plus drive puck, *v* is the velocity just before impact and *d* is the deformation distance (i.e. how much the fur compresses under impact). Thicker fur distributes the impact force over a larger area and longer duration, reducing peak force and adhesion efficiency. For species with thicker fur, researchers may use heavier drive pucks to improve adhesion. However, this must be balanced against the animal’s likely reaction to the impact.

The contact-Japanese Gate, while simpler than the spring-powered version, has the advantage of the animal applying its own force to the tag. Although the exact forces are unknown, preliminary field trials showed this method produced the best tag adhesion on feral dogs. It also eliminates the impact and potential startle reaction of spring-powered systems, as well as the need for complex sensory and tag-projecting equipment. A drawback is that animals may tag themselves on the head, the first body part to pass through the gate. However, animals often lower their heads in restricted spaces [41], a behaviour that can be encouraged with appropriate baiting (electronic supplementary material, film S5).

Increasing the adhesive surface area also improves tag adhesion but reduces the pressure exerted with a standard application force. For very small tags, extending the contact area beyond the tag’s boundaries may enhance adhesion.

Determining the best way to stick tags on to animals is complex. Our attempts to simulate the properties of plant burs were comparatively unsuccessful (cf. figures 7 and 9) given that adhesion forces of some plant burs are substantial. For example, Gorb and Gorb [42] measured separation forces of between 3.3 and 144 mN per individual hook within burs (using four plant species) and noted that, since between 5 and 21 hooks may be involved in binding the fruit to the animal, this equates to total adhesion forces of up to 3 N [42]. These are also presumably dependent on the mammal species used due to differences in the properties of the fur (e.g. figure 8), including in the microstructure of the hairs [43,44]. Studies report that animal hair length affects the number of bur seeds attached [39], which may explain why our measured adhesion forces were greater in furs that had a thicker air layer (figure 7B). Thus, we should expect animals in temperate or polar regions to provide a better adhesion to penetrating bur-based tags than animals in hotter climes [45].

While microstructures provide an adhesive surface, the addition of glues may further improve retention. Plants often use tacky glue to attach burs to animals, and the adhesive secreted by *Pisonia grandis* seeds is so effective at sticking to seabirds that individuals heavily burdened by the burs may be unable to remove them and can die as a result. [46]. We therefore have no doubt that continued research on tacky glues for bur-tagging can produce a step-change in tag-animal binding, particularly if combined with hooks/barbs (see §3.3.1).

We believe that, for now, the best adhesion between tags and their carrier animals will be achieved using natural burs, which have evolved over years to attach effectively to their hosts. These natural burs can be halved, stabilized with glue and attached to the tag after removing the seeds to prevent unwanted species translocation. While many natural burs are designed to detach from the carrier animal after a certain period [21], some species, such as greater burdock can remain attached for weeks or even months (personal observation for domestic sheep *Ovis aries*). Ultimately, assuming that the target animal does not groom the tag off, and a strong bond can be formed between tag and animal (using an adhesive comparable with chewing gum, for example [47]), the tag’s lifespan on the animal could extend to several months. A study that glued tags to polar bears reported a functional life of between 22 and 58 days, depending on tag type [30]. In the best case scenario, this duration would eventually be determined by the moult, which is highly variable between species [48,49].

### 4.4. Animal reaction to the Japanese Gate

#### 4.4.1. Probability that a target animal will interact with the Japanese Gate

A bur-tagging system must include a structure housing the bur tag, strategically placed at a site where the target animal is likely to be tagged. Successful tagging requires the target animal to approach within the operational range of the Japanese Gate. Two factors determine success: (i) the likelihood of the animal coming close enough to the gate, and (ii) whether the gate repels or attracts the animal [50], influenced by species, environmental conditions, learning, memory and timing [51].

Data from camera traps such as we used, in tandem with various factors associated with increased interactions between wildlife and a bur-tagging system (such as baiting [52]), can inform predictions into the probability that the target animal will interact with a Japanese Gate over time. Although there is extensive literature on how the placement of camera traps affects the likelihood of detecting certain species [53,54], much of which seems directly applicable to the bur-tagging system, most camera traps are set to scan a relatively large area (e.g. typically 10−30 m [55]). In contrast, for a successful bur-tagging deployment, the target animal must move within a very well-defined space, in our case moving through the Japanese Gate (see §2.2.1 for dimensions, figure 1). For our purposes, we define that space as the animal being in a position where the sensors within the bur-tagging system would normally initiate tag deployment. The dimensions of this space will depend on the precise set-up used for the bur-tagging system.

**Figure 11.**
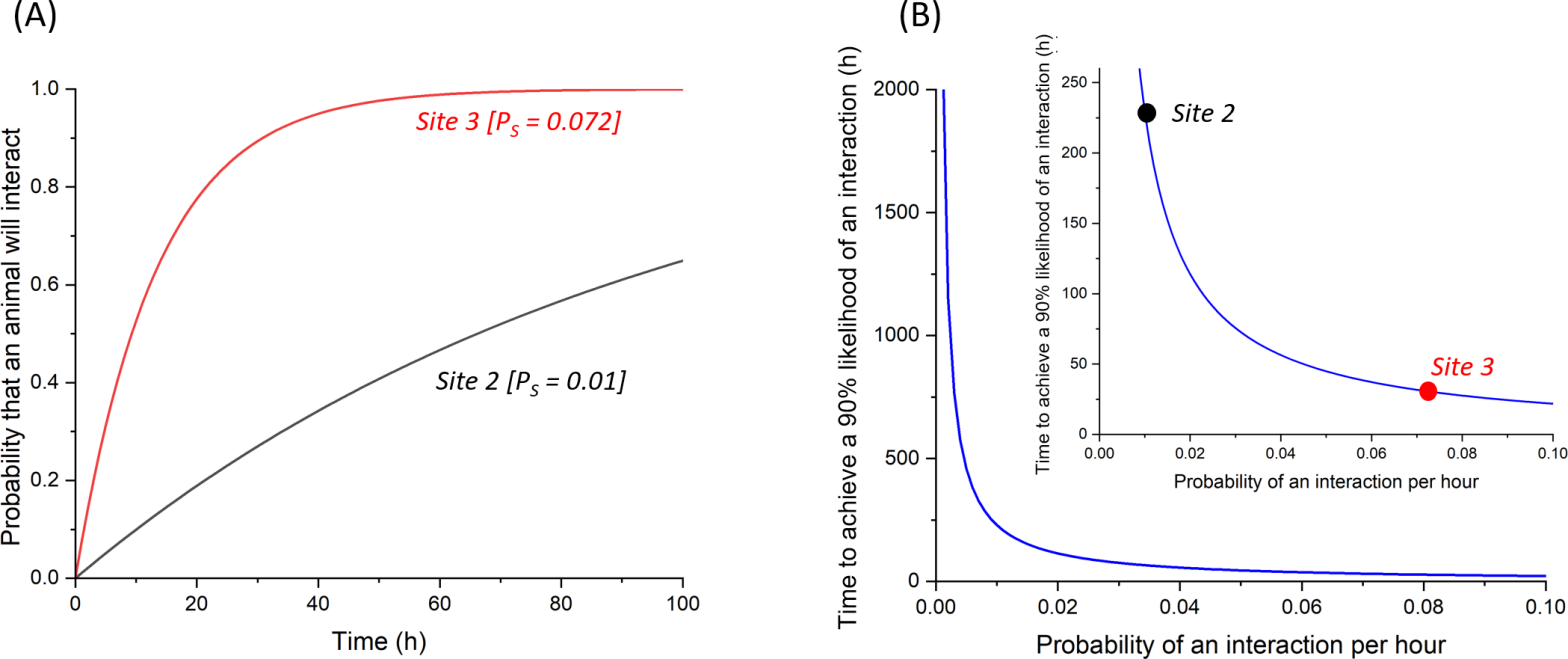
(A) Changing overall probabilities of at least one target animal (here European badgers) being within tagging distance of a Japanese Gate as a function of time according to the incidence of animals seen interacting with the system. Predictions shown are based on camera data from two different sites. Camera data recorded site 2 as having 1 badger interaction over 4 days, the equivalent of a P_s_ of 0.01 h^−1^ (black line). Site 3 recorded 19 interactions over 11 days, which equates to a P_s_ of 0.072 h^−1^ (red line). If this approach is used to calculate the time taken to achieve a 90% likelihood of a badger being within tagging distance of the Japanese Gate, site 2 would need 229 h (black circle in insert in pane B) whereas Site 3 would only need 31 h (red circle).

Camera data on the interaction of target animals with the Japanese Gate can be converted to probabilities that the animals will place themselves in a position to be tagged as a function of system deployment time [56]. This probability (P_Tag_) is critical because, all other things being equal, it affects the expected deployment period before a likely tagging attempt. This can be approximated by standard probability theory whereby the overall probability of an animal being tagged over time,


(5.6) $${\text{P}}_{\text{Tag}}=1-(1-{\text{P}}_{\text{s}}{)}^{\text{t}},$$

where P_s_ is the the probability that the animal will be close enough to the gate to be tagged within a defined time period, and t is the number of time periods over which the bur-tagging system is deployed. The probability that an animal will be close enough to the gate to tag per unit time (P_s_) is derived directly from the camera data and is obtained by dividing the number of instances observed that the target animal came within tagging distance of the gate divided by the duration, ensuring that P_s_ < 1.0 by using an appropriate time period (e.g. hours rather than days). The derived P_s_ value can then be used in equation (5.6). Use of our badger data on interactions between badgers and the Japanese Gates from two different sites, for example, highlights how relatively small changes in P_s_ result in large changes in P_Tag_ with time (figure 11A), particularly when P_s_ is low (figure 11B). Note that this example is generalized and should not be taken to mean that badgers are equally likely to interact with the Japanese Gate during both day and night.

Thus, camera data can be considered together with any information that equates to increased interaction of the target species with the Japanese Gate (such as well-used trails in the right vegetation type, baiting, habitat, etc.) to increase the probability of successful tagging and inform expected outcomes (cf. [57]). This also points to the importance of expert advice in identifying frequently used areas [58]. In addition, given the importance of maximizing P_s_, we would advocate the use of camera traps for sites being considered to help identify well-used sites before deploying any bur-tagging system [59].

### 4.5. Reaction of captive animals and domestic dogs to tag deployment

We believe that remote tag application may cause less stress than traditional methods involving capture, restraint or sedation. However, animals clearly often exhibit neophobia to Japanese Gates, affecting their responses to the bur-tagging process. This sensitivity may increase reactions to stimuli such as servo-motor noise or the tag’s physical impact (cf. [60]).

Our work, while based on captive animals, revealed significant interspecific variation, including social species showing heightened anxiety triggered by tagging-related disturbances (e.g. electronic supplementary material, film S3). Similar patterns were observed in wild animals and are a normal part of stress transmission in group-living mammals [61]. Predictions based on captive studies have limited applicability to wild contexts, so researchers must consider species- and context-specific factors, such as urban versus non-urban animal behaviours, and account for changes as neophobia diminishes over time (figure 10). Camera trapping is essential for documenting these reactions.

Gate design also influences responses. Smaller, more natural gate structures are less likely to provoke avoidance. Zoo trials demonstrated that crossbar height significantly affected species reactions; for example, capybaras (*Hydrochoerus hydrochaeris*) were more disturbed by low-set bars than high-set ones (electronic supplementary material, films S1 and S2). To improve tagging success, bur-tagging systems should minimize their footprint, with sensors and tag dispensers integrated discreetly into the environment, such as by attaching to vegetation and burying cables. Researchers should also wear gloves to reduce olfactory cues (cf. [62]).

### 4.6. Preliminary field trials

This fieldwork indicates that it is possible to tag animals (feral dogs in our case) using our system for periods of hours at least and we consider that the well-embedded tag seen on the one relocated animal would have lasted at least a few days. Certainly, greater burdock burs that have latched on to domestic sheep can remain for weeks or even months (personal observations) and can be so persistent that they can eventually lead to the death of smaller mammals and birds ([63] and references therein, [64]).

### 4.7. Limitations

#### 4.7.1. Tag recovery

This study did not focus on tag retention or data recovery, both of which are important elements of bur-tagging. An obvious way to retrieve data from a bur tag is through Bluetooth or VHF, offering convenience but limited range [65], although larger tags could use satellite transmission [66]. When physical recovery is required, retrieving shed bur tags may be easier than recapturing the animal. Automatic drop-off systems, common in collar-mounted tags [67], simplify this process and might conceivably be used in bur-tagging applications if only one side of the tag is adhesive. Shed or automatically released bur tags could be recovered using a homing beacon, such as a VHF transmitter [68], as demonstrated effectively in our trials.

#### 4.7.2. Tag dispensing

Our current system only dispenses a single tag before manual intervention is needed for reloading. A huge advance would be to have a system with the option to dispense multiple tags. This would require careful engineering but would be particularly advantageous if used at sites frequented by many individuals, such as a waterhole. In tandem with this, AI could be used for proper identification of the target species as well as to control the trajectory of the tag (including through movement of the dispenser nozzle) so that it landed perfectly placed. All this would increase the potential of the system as well as minimizing the inadvertent tagging of non-target species, simplifying ethical issues and increasing the quality of the data gained.

#### 4.7.3. Tag ingestion

Tags removable by animals carry a risk of ingestion. While we observed no such behaviour in trials, including with domestic dogs, and could find no data on this in the literature on land mammals, this possibility should be addressed. Similarly, tags can potentially be ingested by biologged animals’ predators (e.g. [69]). To minimize the potential for harm, tags could be coated with unpalatable substances [70] or designed to pass harmlessly through the digestive system if ingested. Such tags must be small enough to pass safely, and robust enough to resist damage during mastication.

## 5. Conclusion

To enhance animal welfare, we recommend that researchers consider a bur-tagging approach for free-living species. Properly executed, bur-tagging may cause only marginally more stress than natural burs, making even short deployments beneficial and ethically defendable [71]. As tags become smaller, cheaper and more lightweight [72], the case for bur-tagging grows stronger, potentially allowing multiple tags on a single animal, similar to vegetation burs [73].

Our current system dispenses single tags from a stationary unit with a basic sensor for detecting animal type and position. However, advances in AI could enable future systems to deploy tags serially, identify species and sex, and optimize tag placement through directed dispensers. Although our work is geared towards animals with fur, in theory and with suitable attachment mechanisms, there is no reason why similar systems should not work on reptiles, birds and even marine animals such as fish. While our study is preliminary, it highlights the potential of bur-tagging and the critical design decisions required, such as sensor types, tag deployment methods and adhesion mechanisms. Documenting animal reactions and tagging processes with cameras can guide further development.

We hope this pilot work inspires researchers to explore bur-tagging for their study species. As the technique evolves, it promises to improve both the welfare of tagged animals and the quality of data collected in wildlife research.

## Data Availability

Original data is available online [74]. Supplementary material is available online [75].
